# *KMT2D* mutations promoted tumor progression in diffuse large B-cell lymphoma through altering tumor-induced regulatory T cell trafficking via FBXW7-NOTCH-MYC/TGF-β1 axis

**DOI:** 10.7150/ijbs.93349

**Published:** 2024-07-15

**Authors:** Qing-Xiao Liu, Yue Zhu, Hong-Mei Yi, Yi-Ge Shen, Li Wang, Shu Cheng, Peng-Peng Xu, Hai-Min Xu, Lu-Ting Zhou, Yao-Hui Huang, Chuan-Xin Huang, Di Fu, Meng-Meng Ji, Chao-Fu Wang, Wei-Li Zhao

**Affiliations:** 1 Shanghai Institute of Hematology, State Key Laboratory of Medical Genomics, National Research Center for Translational Medicine at Shanghai, Ruijin Hospital, Shanghai Jiao Tong University School of Medicine, Shanghai 200025, China.; 2 Department of Pathology, Ruijin Hospital, Shanghai Jiao Tong University School of Medicine, Shanghai 200025, China.; 3 Department of Immunobiology and Microbiology, Shanghai Institute of Immunology, Shanghai Jiao Tong University School of Medicine, Shanghai 200025, China.; 4 Pôle de Recherches Sino-Français en Science du Vivant et Génomique, Laboratory of Molecular Pathology, Shanghai 200025, China.

**Keywords:** diffuse large B-cell lymphoma, KMT2D, regulatory T cells, FBXW7, NOTCH, MYC, TGF-β1

## Abstract

Histone methyltransferase *KMT2D* is one of the most frequently mutated genes in diffuse large B-cell lymphoma (DLBCL) and has been identified as an important pathogenic factor and prognostic marker. However, the biological relevance of *KMT2D* mutations on tumor microenvironment remains to be determined. *KMT2D* mutations were assessed by whole-genome/exome sequencing (WGS/WES) in 334 patients and by targeted sequencing in 427 patients with newly diagnosed DLBCL. Among all 761 DLBCL patients, somatic mutations in *KMT2D* were observed in 143 (18.79%) patients and significantly associated with advanced Ann Arbor stage and MYC expression ≥ 40%, as well as inferior progression-free survival and overall survival. In B-lymphoma cells, the mutation or knockdown of *KMT2D* inhibited methylation of lysine 4 on histone H3 (H3K4), downregulated FBXW7 expression, activated NOTCH signaling pathway and downstream MYC/TGF-β1, resulting in alterations of tumor-induced regulatory T cell trafficking. In B-lymphoma murine models established with subcutaneous injection of SU-DHL-4 cells, xenografted tumors bearing *KMT2D* mutation presented lower H3K4 methylation, higher regulatory T cell recruitment, thereby provoking rapid tumor growth compared with wild-type *KMT2D* via FBXW7-NOTCH-MYC/TGF-β1 axis.

## Introduction

Diffuse large B-cell lymphoma (DLBCL) is a molecularly heterogeneous entity of non-Hodgkin lymphoma with variations in genetic alterations, leading to considerable differences in clinical course and response to immunochemotherapy 1. Two-thirds of DLBCL patients are curable with standard R-CHOP (rituximab plus cyclophosphamide, doxorubicin, vincristine, and prednisone), while the remaining patients are refractory or relapsed from immunochemotherapy, urging targeted therapeutic approaches to be further investigated 2.

With the development of next-generation sequencing technologies, remarkable progress has been made to illustrate the oncogenic mechanisms in DLBCL progression 3. Epigenetic gene alterations are major pathogenic factors of DLBCL 1. Among these genes, the most frequently mutated gene *KMT2D* (also known as *MLL2*/*MLL4*) encodes a highly conserved protein that catalyzes the methylation of lysine 4 on histone H3 (H3K4) 4. The enzymatic function of KMT2D depends on a cluster of C-terminal conserved domains, including a PHD domain, two FY-rich motifs (FYRC and FYRN) and a catalytic SET domain 5. Most *KMT2D* mutations are nonsense or frameshift mutations, which are likely to confer loss of KMT2D function and diminished global H3K4 methylation 1. Lack of functional KMT2D promotes lymphomagenesis in murine models and inactivation of KMT2D activates oncogenic signaling pathways such as NOTCH, RAS, and ERK 4, 6, 7.

Tumor microenvironment facilitates malignant cell escape from immune cells and subsequently provokes tumor progression in DLBCL 8, 9. Multiple mechanisms lead to immunosuppressive conditions, including impaired functions of effector T and natural killer cells, as well as induction of regulatory T (Treg) cells, myeloid-derived suppressor cells, and tumor-associated macrophages 10, 11. Particularly, Treg cells play an essential role in immune homeostasis 12, 13, dividing by natural Treg cells developed in the thymus and inducing Treg cells differentiation from naïve CD4^+^ T cells at the periphery 14, 15. Tumor induced Treg cell trafficking into microenvironment ensures efficient immune response. However, the potential effect of *KMT2D* mutations on tumor microenvironment remains to be determined in DLBCL. Here we performed the genomic and transcription analysis in a large cohort of DLBCL patients and further revealed the effect of *KMT2D* mutations on lymphoma microenvironment. Meanwhile, underlying molecular mechanisms of *KMT2D* mutations on tumor-induced Treg cell trafficking were investigated both *in vitro* and *in vivo*.

## Materials and methods

### Patients

In all, 761 patients with newly diagnosed DLBCL were included in this study. Two experienced pathologists (YHM and WCF) established histological diagnosis according to the World Health Organization classification. All patients were treated with R-CHOP-based immunochemotherapy. The treatment response was evaluated according to the International Workshop Criteria. The Hospital Review Board approved the study with informed consent obtained following the Declaration of Helsinki.

### Cell lines and reagents

B-lymphoma cell line SU-DHL-4 (obtained from American Type Culture Collection, Manassas, VA, USA) and U-2932 (obtained from Deutsche Sammlung von Mikroorganismen und Zellkulturen, Braunschweig, Lower Saxony Land, Germany) were grown in Roswell Park Memorial Institute (RPMI)-1640 medium, supplemented with 10% heat-inactivated fetal bovine serum and 1% penicillin/streptomycin (15140122, Gibco, Carlsbad, CA, USA) in a humidified atmosphere containing 95% air-5% CO2 at 37°C. γ-secretase inhibitor (inhibitor of the NOTCH signaling pathway, GSI-I, S1575) and MYC inhibitor (S7153) were purchased from Selleck (Houston, TX, USA).

### DNA sequencing

Tumor samples of 761 patients were analyzed for gene mutations using whole genome sequencing/whole exome sequencing (WGS/WES) or targeted sequencing. WGS (n=109) was performed on frozen tumor tissue. WES (n=225) was performed on frozen tumor tissue and formalin-fixed paraffin-embedded tumor tissue quality-controlled by agarose gel electrophoresis. Targeted sequencing (n=427) was performed on frozen tumor tissue. Mutation frequencies per gene and mutation signatures showed no significant difference in the results for WGS, WES and targeted sequencing.

### RNA sequencing

Total RNA was extracted from tumor samples of 402 DLBCL patients. RNA was purified using Ribo-Zero rRNA Removal Kits (Illumina). RNA concentration and integrity were verified using NanDrop and Agilen 2100 Bioanalyzer, respectively. RNAs libraries were constructed with TruSeq RNA Library Preparation Kit (Illumina). The concentration and the quality of libraries were controlled by Qubit and BioAnalyzer 2100 system. The paired-end sequencing was performed on Illumina HiSeq Sequencer. After 3' adaptor-trimming and removing low-quality reads, high quality trimmed reads were aligned to the reference genome (UCSC hg19). Pathway enrichment analysis was performed based on the differentially expressed mRNAs through the user tutorials of Cytoscape referring to Kyoto Encyclopedia of Genes and Genomes (KEGG) databases. Gene Set Enrichment Analysis (GSEA) was performed using the BROAD Institute GSEA software.

### Cell transfection

For Vector, *KMT2D*^wt^, *KMT2D*^C5092R^, *KMT2D*^G5182fs^, *KMT2D*^W5395X^, *KMT2D*^R5432Q^, *KMT2D*^kd^ and Scramble transfection, purified plasmids pGV358/GFP/Puro (Vector), pGV358/GFP/Puro-*KMT2D* (NM-003482, residues 4839-5537, containing SET domain, wild-type, *KMT2D*^C5092R^, *KMT2D*^G5182fs^, *KMT2D*^W5395X^ and *KMT2D*^R5432Q^), pGV248/GFP/Puro (Scramble), pGV248/GFP/Puro-sh* KMT2D* were transfected into packages HEK-293T cells using lipofectamine 2000 (11668019, Invitrogen, Carlsbad, CA, USA) according to the manufacturer's protocol. The supernatant fraction of HEK-293T cell cultures was then condensed to a viral concentration of approximately 2 × 10^8^ transducing units/ml. The lentiviral particles were incubated with SU-DHL-4 or U-2932 cells for 72h with addition of polybrene (8μg/ml). The stably transduced clones were selected by green fluorescence protein using flow cytometry or puromycin treatment for two weeks. The shRNA sequences of *KMT2D* were listed in Table. S1.

### Western blot

Cells were lysed in 200μl lysis buffer (0.5M Tris-HCl, pH 6.8, 2mM EDTA, 10% glycerol, 2% SDS and 5% β-mercaptoethanol). Protein lysates (20μg) were electrophoresed on 10% SDS polyacrylamide gels and transferred to nitrocellulose membranes. Membranes were blocked with 5% non-fat dried milk and incubated overnight at 4°C with appropriate antibodies, followed by a horseradish peroxidase-conjugated secondary antibody. The immunocomplexes were visualized using a chemiluminescence phototope-horseradish peroxidase Kit (Cell Signaling Technologies, Danvers, MA, USA). Primary antibodies included H3K4me3 (ab8580, Abcam, Cambridge, United Kingdom), FBXW7 (28424-1-AP, Proteintech), NICD (ab8925, Abcam), MYC (ab32072, Abcam), TGF-β1 (ab215715, Abcam). Histone 3 (17168, Proteintech) and β-Actin (ab8226, Abcam) were used to ensure equivalent loading of protein. Horseradish peroxidase-conjugated secondary antibodies against goat anti-mouse-IgG and goat anti-rabbit-IgG were from Cell Signaling Technologies.

### Immunohistochemistry and immunofluorescence assay

Immunohistochemistry was performed on 5μm-paraffin sections with an indirect immunoperoxidase method using antibodies against MYC (1:500, ab32072, Abcam), H3K4me3 (1:1000, ab8580, Abcam), intracellular portions of NOTCH1 (NICD, 1:200, ab8925, Abcam) and Foxp3 (1:500, ab20034, Abcam). H3K4me3 and NICD expression levels were scored based on the percentage of positive cells. For Foxp3, immunoreactivity in > 0% of cells was defined as positive 16. Immunofluorescence assay of H3K4me3 was performed on acetone-fixed cells, using rabbit anti-H3K4me3 as primary antibodies and Alexa Fluor 594-conjugated goat polyclonal anti-rabbit IgG-H&L (ab150080, Abcam) as the secondary antibody. Nuclei were counterstained with DAPI.

### Quantitative real-time PCR (RT-PCR)

Total mRNA was extracted using Trizol reagent and reverse transcribed using a PrimeScript RT Reagent Kit with gDNA Eraser for quantitative RT-PCR (RR047A, TaKaRa, Japan). Quantitative RT-PCR was performed by SYBR Premix Ex TaqTM II (RR820A, TaKaRa) and ABI ViiA7 (Applied Biosystems, Bedford, MA, USA) with primers against TGF-β1 and FBXW7. Relative quantification was calculated using the 2-∆∆CT methods. The primers were listed in Table. S2.

### Chromatin immunoprecipitation (ChIP)

Nuclear extracts were prepared from 2 × 10^7^ cells per sample. Rabbit anti-human H3K4me3 antibody (ab8580, Abcam) was used for immunoprecipitation, and normal IgG (3900, Cell Signaling Technologies) was referred as negative control. ChIP primers of *FBXW7* genes were used as previously reported 17, which were designed to detect promoter fragments near transcription start sites. ChIP-enriched chromatin was used for real-time PCR with SYBR Premix Ex TaqTM II, normalizing to input.

### B-lymphoma cell co-culture

Co-culture of SU-DHL-4 or U-2932 cells with PBMCs was conducted by a 0.4μm pore polycarbonate membrane and 6.5mm inserts (Corning, Sunnyvale, CA, USA). SU-DHL-4 and U-2932 cells transfected with Vector, *KMT2D*^wt^, *KMT2D*^C5092R^, *KMT2D*^G5182fs^, *KMT2D*^W5395X^, *KMT2D*^R5432Q^, Scramble, and *KMT2D*^kd^ were cultured at 2 × 10^5^ cells/ml in the upper chamber, while PBMCs at 1 × 10^6^ cells/ml (1:5 ratio) in the lower chamber. Cell viability was assessed by CCK8. After 72h co-culture, we measured its absorbance at 450nm by spectrophotometry. All cells were maintained in RPMI-1640 medium supplemented with 10% heat-inactivated fetal bovine serum and 1% penicillin/streptomycin.

### Single-cell RNA sequencing

Tumor samples of six DLBCL patients were collected for single-cell RNA sequencing analysis. Tumor samples were placed in separate containers containing Tissue Storage Solution (Miltenyi Biotec) and transported in regular ice to the laboratory immediately. Tumor samples were separated into small pieces mechanically and dissociated into single-cells after enzymatic digestion at 37 ℃. After filtering with a 40-µm strainer and washing once with PBS, cells were cryopreserved in liquid nitrogen before the scRNA-seq experiment. For the scRNA-seq experiment, cells were thawed and washed immediately, and further processed with Dead cell removal kit (Miltenyi Biotec). The preparation of the single-cell suspensions, synthesis of complementary DNA and gene expression libraries were performed according to the manufacturer's instructions using Chromium single cell 3' Kit v2 (10x Genomics) in 2 patients, and Chromium single cell 5' Kit v2 (10x Genomics) in 4 patients. The 3' gene expression libraries were sequenced on Novaseq 6000 (Illumina) and 5' gene expression libraries on MGI-2000 sequencer.

Reading the raw data by the Read10X function in the seurat package (4.0.2) 18. The data filtering criteria were: minimum number of cells was 3, minimum number of genes tested was 300, and the percentage of mitochondrial gene expression was less than 5%. The data were first normalized by LogNormalize method, and the top 1500 genes with large intercellular variation coefficients were extracted by FindVariableFeatures function. The normalized count matrix is linearly transformed ("scaling") by the ScaleData function to normalize the data: 1) shifting the expression of each gene so that the average expression between cells is 0; 2) scaling the expression of each gene so that the difference between cells to 1. The normalized expression matrix was subjected to PCA downscaling using the RunPCA function.

The distribution of p-values for each PC was calculated using the JackStraw function. Significant PCs would have lower p-values, and the appropriate PC values were filtered. The above descended data were clustered by t-distributed stochastic neighbor embedding (tSNE) algorithm, and the differential gene analysis was performed by FindAllMarkers function for each subpopulation to filter Marker genes. The subpopulations were annotated according to marker genes by SingleR package (1.4.1) 19, in which the annotation comparison data set was HumanPrimaryCellAtlasData, and the similarities and differences of cell numbers between cancer and normal groups were counted after subpopulation annotation. The expression matrices of T cells from patients with or without *KMT2D* mutations were extracted separately and merged by merge function. The differential expression genes of T cells were calculated by FindAllMarkers function. The screening criteria for differentially expressed genes were P value < 0.05.

### Flow cytometry

Antibodies used for cell labeling of Treg cells were as followed: BV421 anti-CD4 (562424, BD Biosciences), BV786 anti-CD25 (563701, BD Biosciences), PE anti-Foxp3 (560046, BD Biosciences). Flow cytometry data were collected by a FACS Calibur cytometer (BD Biosciences) and analyzed by FlowJo software.

### Luciferase report assay

Total cDNA from HEK-293T cells was used to amplify the promoter (-1914 to +79 bp) of TGF-β1, forward primer: 5′-GCCCGCAACATATAGATGAGGACGGTGGCCCAGCCC-3′; reverse primer: 5′-CTATATGTTGCGGGCTCCGAGGGGGGTC-3′. After digestion with BamHI and EcoRI, PCR products were ligated into the GM-4629: PGL3-basic vector and confirmed by DNA sequencing. HEK-293T cells were seeded in 24-well plates and co-transfected with 250ng of MYC, 250ng promoter (-1914 to +79 bp) luciferase reporter construct and 50μl luciferase reporter. Cells were collected 24h after transfection, using the Cell Lysis Buffer (100μl per well) provided as part of the Dual-Luciferase Reporter Assay System Kit (Promega, Madison, WI, USA). Firefly and Renilla luciferase activities were examined by the Dual-Luciferase Reporter Assay System and detected by a Centro XS3 LB960 Luminometer (Berthold, Bad Wildbad, Germany).

### Mice

NOD-*Prkdc*^scid^*Il2rg*^em1^/Smoc (M-NSG) immune deficient female mice were used in this study, which purchased from Shanghai Model Organisms, ages 6 to 8 weeks at experiment initiation. M-NSG mice were injected subcutaneously with *KMT2D*^wt^ and *KMT2D*^R5432Q^ SU-DHL-4 cells (1 × 10^7^ cells) and human PBMC intravenously (5 × 10^6^ cells). Tumor volumes were measured twice a week and human CD45 (hCD45) were measured after 3 weeks to confirm that humanized PBMCs were established. TGF-β inhibitor (purchased from Selleck, SB431542) was injected intraperitoneally in *KMT2D*^R5432Q^ mice at a dose of 10mg/kg per week, 7 days after tumor cell injection. Tumor volumes were calculated at 0.5 × a (length) × b (width)^2^. Animals were used according to the protocols approved by the Shanghai Rui Jin Hospital Animal Care and Use Committee.

### Statistical analysis

The baseline characteristics of patients were measured by χ2 test. Progression-free survival (PFS) was calculated from the date when treatment began to the date when the disease progression was recognized or the date of last follow-up. Overall survival (OS) was measured from the date of diagnosis to the date of death or last follow-up. Univariate hazard estimates were generated with unadjusted Cox proportional hazards models. Survival functions were estimated using the Kaplan-Meier method and compared by the log-rank test. Experimental results were calculated as the mean ± standard deviation from three separate experiments. The student t-test was applied to compare two normally distributed groups and Mann-Whitney U test to compare which did not conform to normal distribution. All statistical analysis was carried out using Statistical Package for the Social Sciences (SPSS, 26.0) software or GraphPad Prism 8 software. Statistical significance was defined as p < 0.05.

## Results

### *KMT2D* mutations contributed to tumor progression and aberrant tumor microenvironment in DLBCL

**Figure 1:**
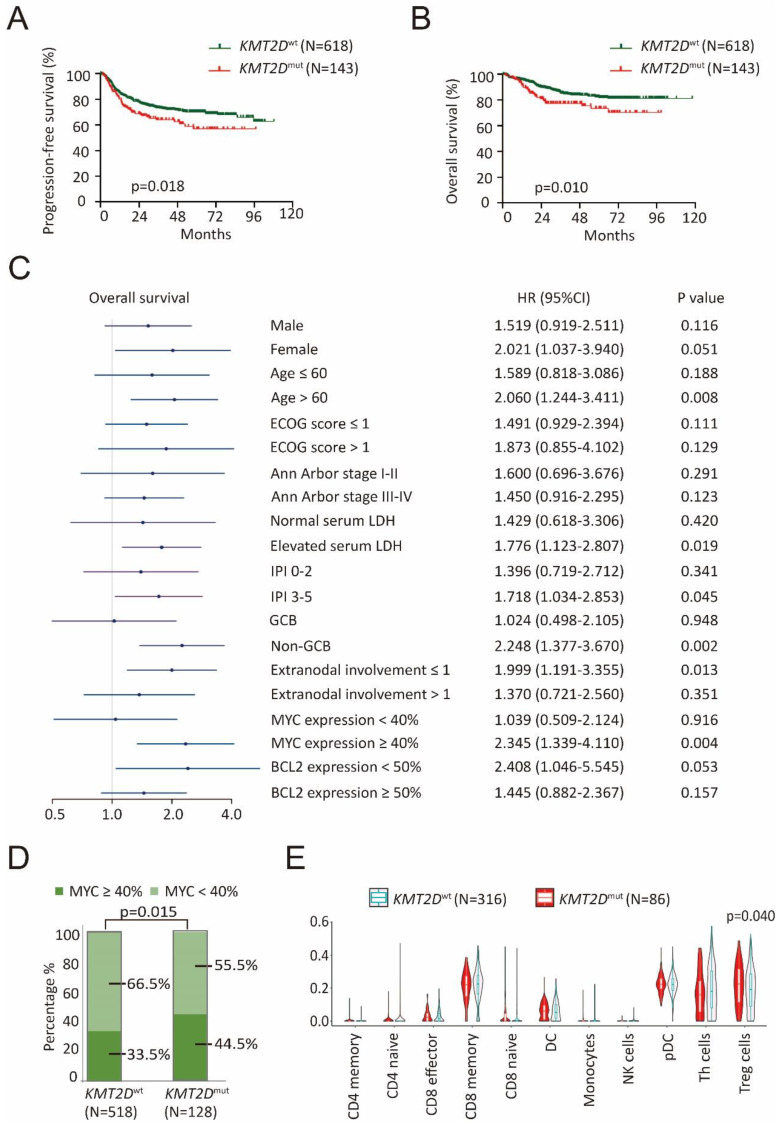
*** KMT2D* mutations contributed to tumor progression and aberrant tumor microenvironment in diffuse large B-cell lymphoma (DLBCL). (A). PFS (progression free survival) curves of *KMT2D*^wt^ and *KMT2D*^mut^ DLBCL patients. (B). OS (overall survival) curves of *KMT2D*^wt^ and *KMT2D*^mut^ DLBCL patients.** (C). Forest plot of univariate analysis on overall survival (OS) of selected subgroups. (D). Immunohistochemistry study of MYC in tumor samples of DLBCL patients with or without *KMT2D* mutations. (E). TIP analysis showing association of Treg cell gene signatures with *KMT2D* mutations in DLBCL patients.

A total of 761 patients with newly diagnosed DLBCL were analyzed by WGS/WES and targeted sequencing, including 618 patients with wild-type *KMT2D* (*KMT2D*^wt^) and 143 patients with *KMT2D* mutations (*KMT2D*^mut^). The clinical characteristics of these patients were summarized in Table 1. *KMT2D*^mut^ patients were significantly associated with advanced Ann Arbor stage (III-IV, p=0.003) and MYC expression ≥ 40% (p=0.015), as compared to *KMT2D*^wt^ patients. The median follow-up time was 38.4 months (range: 0.3-118.1 months). Inferior progression-free survival (PFS) and overall survival (OS) were presented in *KMT2D*^mut^ patients (p=0.018, Figure 1A and p=0.010, Figure 1B). Moreover, inferior OS was observed in *KMT2D*^mut^ patients with age > 60 years, elevated serum lactate dehydrogenase (LDH), International Prognostic Index (IPI) 3-5, non-germinal center B-cell-like (GCB) subtype, extranodal involvement ≤ 1 and MYC expression ≥ 40% (p=0.008, p=0.019, p=0.045, p=0.002, p=0.013 and p=0.004, respectively, Figure 1C). Inferior PFS was observed in *KMT2D*^mut^ patients with age > 60 years, ECOG score > 1, IPI 3-5, non-GCB subtype, extranodal involvement ≤ 1, MYC expression ≥ 40% and BCL2 expression < 50% (p=0.012, p=0.008, p=0.028, p=0.012, p=0.030, p=0.033 and p=0.040, respectively, Supplementary Figure 1). *KMT2D*^mut^ patients presented higher incidence of MYC expression ≥ 40%, as compared to *KMT2D*^wt^ patients (p=0.015, Figure 1D).

To explore the potential role of *KMT2D* mutations on tumor microenvironment, RNA sequencing was performed on 402 patients, including 86 patients with *KMT2D* mutations and 316 patients with wild-type *KMT2D*. Within several innate and adaptive immune cell subsets, Treg cell infiltration was significantly associated with *KMT2D* mutations (p=0.040, Figure 1E), as revealed by tracking tumor immunophenotype (TIP) analysis 20. Meanwhile, gene sets that present immunologic cells were employed in GSEA (Molecular signatures database C7: immunologic signature gene sets). Indeed, comparing *KMT2D*^mut^ with *KMT2D*^wt^ patients, significant activation of Treg cells was observed by GSEA (Supplementary Figure 2) 21.

### *KMT2D* mutations inhibited H3K4 methylation and induced Treg cells with tumor microenvironment

**Figure 2:**
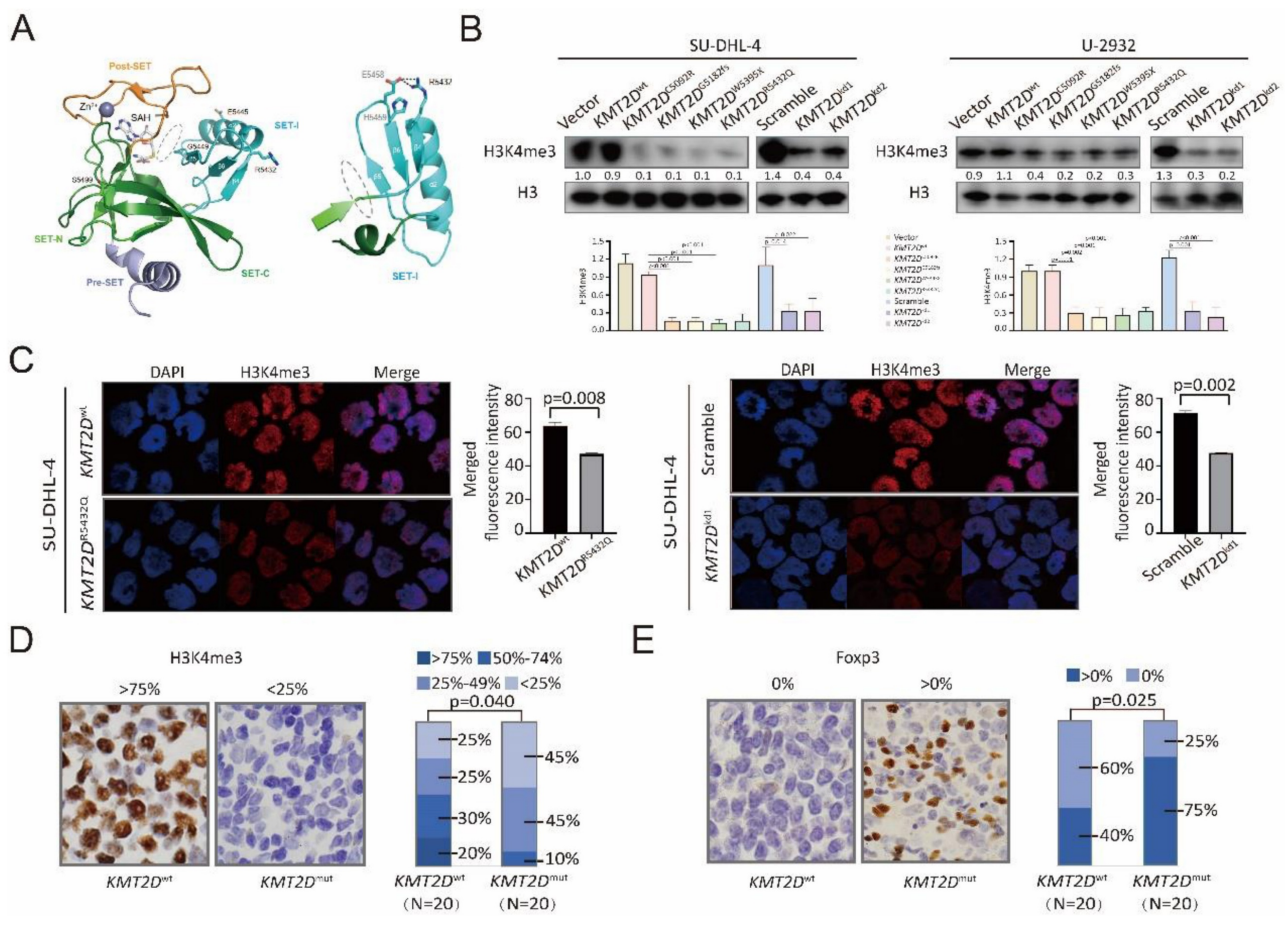
*** KMT2D* mutations inhibited H3K4 methylation and induced Treg cells.** (**A**). Cartoon representation of KMT2D (PDB 4Z4P). The cofactor byproduct S-adenosyl-L-homocysteine (SAH) and mutations (R5432Q, E5445A, G5449S and S5499R) are shown as sticks. The coordinated Zn^2+^ ion shown as a sphere. The H3K4me binding channel is indicated as gray oval (left panel). Cartoon representation of SET-I. The orientation of the SET-I region is important for KMT2D activation, as SET-I and Post-SET domains anchor H3K4me substrate promoting its proximity to the SAH and active sites. The position of β-sheet 4 and 6 are stabilized by R5432, E5458 and H5459, R5432 forms salt bridges with E5458, H5459 assisted the salt bridge stability. R5432Q mutation disordered the SET-I region stability causing H3K4me docking into binding channel difficulty. Consequently, R5432Q mutation shown reduced methyltransferase activity compared with wild-type (right panel). (**B**). Protein expression of H3K4me3 detected in Vector,* KMT2D*^wt^, *KMT2D*^C5092R^, *KMT2D*^G5182fs^, *KMT2D*^W5395X^, *KMT2D*^R5432Q^, Scramble, *KMT2D*^kd1^, *KMT2D*^kd2^ of SU-DHL-4 and U-2932 cells by western blot. H3 was used as loading controls. (**C**). Immunofluorescence assay of H3K4me3 in tumor samples of *KMT2D*^wt^, *KMT2D*^R5432Q^, Scramble and *KMT2D*^kd1^of SU-DHL-4 cells. (**D, E**). Immunohistochemistry study of H3K4me3 (**D**) and Foxp3 (**E**) in tumor samples of DLBCL patients with and without *KMT2D* mutations.

The possible structure-function relationship of the mutants was addressed using crystal structure of the protein encoded by KMT2D (PDB 4Z4P). For example, KMT2D C5092 is Zinc-coordinating cysteine on PHD domain, C5092R affected KMT2D binding to methylated histones and other nuclear proteins. KMT2D G5182fs and W5395X caused premature termination of translation, resulting in functional haploinsufficiency. KMT2D R5432Q reduced methyltransferase activity by disordering the SET-I region stability causing H3K4me docking into binding channel difficulty, as compared to wild-type KMT2D (Figure 2A and Supplementary Figure 3). To determine the biological function of *KMT2D* mutations, germinal center B-cell (GCB) DLBCL cell line (SU-DHL-4) and activated B-cell (ABC) DLBCL cell line (U-2932) were engineered to express a fragment of KMT2D (amino acid 4839-5537, containing SET domain for wild-type, *KMT2D*^C5092R^, *KMT2D*^G5182fs^, *KMT2D*^W5395X^ and *KMT2D*^R5432Q^) protein. *KMT2D*^wt^, *KMT2D*^C5092R^, *KMT2D*^G5182fs^, *KMT2D*^W5395X^ and *KMT2D*^R5432Q^, as well as shRNA to knockdown *KMT2D* (*KMT2D*^kd^) were established and transfected into SU-DHL-4 and U-2932 cells. *KMT2D* encodes a highly conserved protein that catalyzes the methylation of lysine 4 on histone H3 (H3K4) 6. Compared with Vector or Scramble cells, the trimethylation of lysine 4 on histone H3 (H3K4me3) levels were decreased in *KMT2D*^C5092R^, *KMT2D*^G5182fs^, *KMT2D*^W5395X^, *KMT2D*^R5432Q^, or* KMT2D*^kd^ SU-DHL-4 and U-2932 cells, as revealed by western blot (Figure 2B). Representative immunofluorescence assay of H3K4me3 expression was shown in *KMT2D*^R5432Q^ and* KMT2D*^kd^ SU-DHL-4 cells. Nuclei were counterstained with DAPI. Fluorescence intensity of H3K4me3 represents protein expression level.

According to the merged fluorescence intensity of DAPI and H3K4me3, the H3K4me3 expression in *KMT2D*^R5432Q^ and* KMT2D*^kd^ SU-DHL-4 cells was higher than *KMT2D*^wt^ and Scramble SU-DHL-4 cells (Figure 2C). In consistence with *in vitro* data, a lower percentage of nuclear H3K4me3-positive cells was observed in tumor samples of *KMT2D*^mut^ patients than those of *KMT2D*^wt^ patients (p=0.040, Figure 2D). Higher levels of Treg cells, characterized by Foxp3-positive cells, were observed in tumor samples of *KMT2D*^mut^ patients than those of *KMT2D*^wt^ patients (p=0.025, Figure 2E). Therefore, *KMT2D* mutations were biologically functional and related to alterations in tumor-induced Treg cells in DLBCL.

### *KMT2D* mutations downregulated *FBXW7* and activated NOTCH/MYC signaling

**Figure 3:**
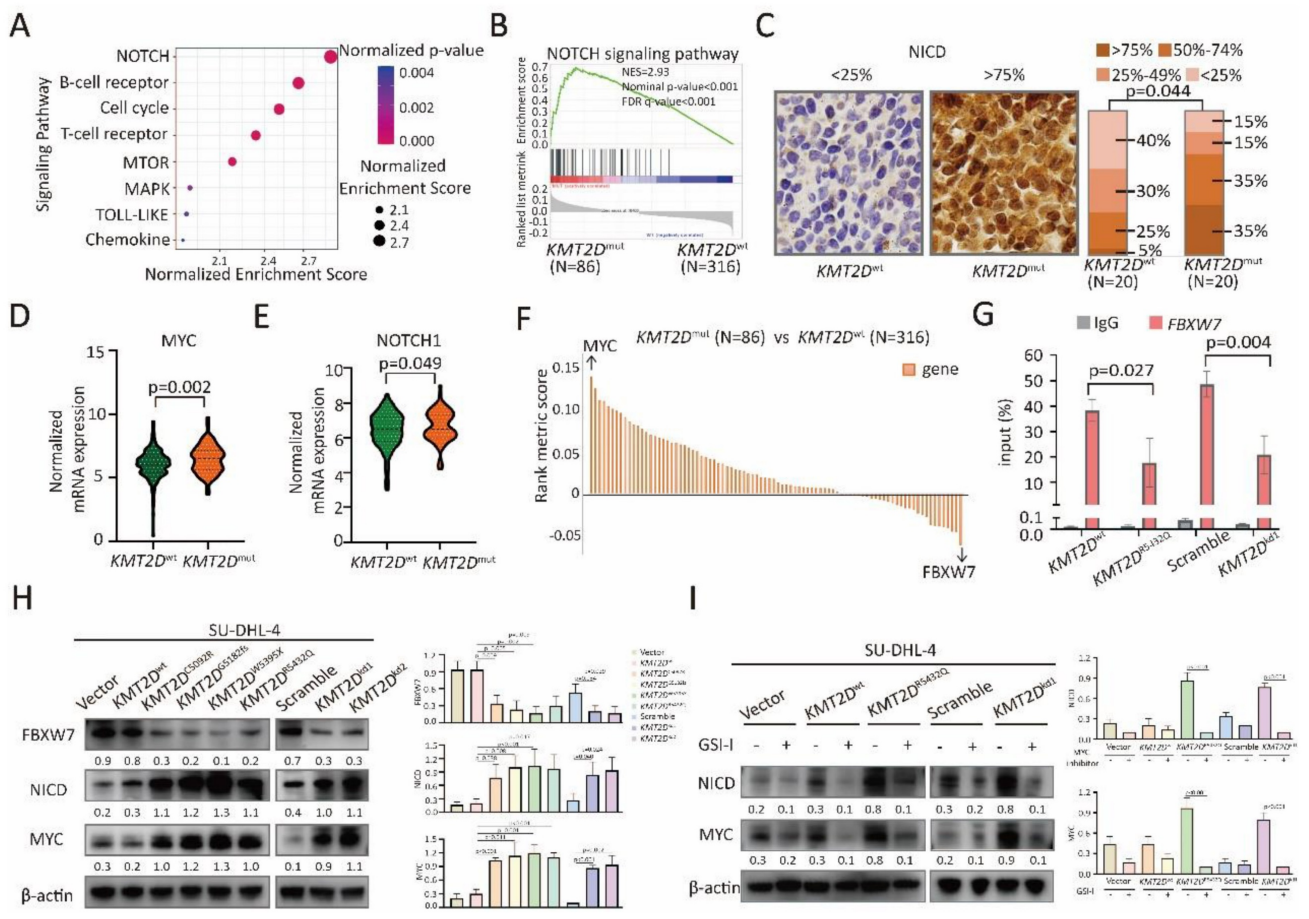
*** KMT2D* mutations inhibited FBXW7 and activated NOTCH/MYC cascade.** (**A**). Pathway enrichment analysis of DLBCL patients with or without *KMT2D* mutations according to the Kyoto Encyclopedia of Genes and Genomes (KEGG) database. (**B**). Gene Set Enrichment Analysis (GSEA) enriched differentially expressed genes in NOTCH signaling pathway with or without *KMT2D* mutations. Enrichment scores were listed with p-value. NES, normalized enrichment score; FDR, false discovery rate. (**C**). Immunohistochemistry study of intracellular portions of NOTCH1 (NICD) in tumor samples of DLBCL patients with or without *KMT2D* mutations. (**D**). Normalized mRNA expression of MYC in tumor samples of DLBCL patients with or without *KMT2D* mutations as revealed by RNA sequencing. (**E**). Normalized mRNA expression of NOTCH1 in tumor samples of DLBCL patients with or without *KMT2D* mutations as revealed by RNA sequencing. (**F**). Genes associated with NOTCH signaling pathway calculated based on rank metric score from GSEA in *KMT2D*^mut^ DLBCL patients, as compared to *KMT2D*^wt^ DLBCL patients*.* (**G**). Occupancies of H3K4me3 in the proximal promoter areas of *FBXW7* in *KMT2D*^wt^, *KMT2D*^R5432Q^, *KMT2D*^kd^ and Scramble SU-DHL-4 cells by chromatin immunoprecipitation (ChIP) assay. (**H**). Protein expression of FBXW7, NICD and MYC in *KMT2D*^C5092R^, *KMT2D*^G5182fs^, *KMT2D*^W5395X^, *KMT2D*^R5432Q^, *KMT2D*^kd1^ and *KMT2D*^kd2^ SU-DHL-4 cells, as compared to *KMT2D*^wt^ or Scramble cells by western blot. β-actin was used as a loading control. (**I**). Protein expression of NICD and MYC in *KMT2D*^R5432Q^ and *KMT2D*^kd^ SU-DHL-4 cells by western blot with and without NOTCH inhibitor (γ-secretase inhibitor, GSI-I, 50μm) treatment for 48h. β-actin was used as a loading control.

To determine the possible effects of *KMT2D* mutations on signaling transduction, RNA sequencing was performed on 402 patients, and differentially expressed genes were analyzed by KEGG. Modulations of multiple signaling pathways were identified (Figure 3A), particularly NOTCH signaling pathway (p < 0.001, Figure 3B). A higher percentage of intracellular portions of NOTCH1 (NICD)-positive cells were observed in tumor samples of *KMT2D*^mut^ patients than those of *KMT2D*^wt^ patients (p=0.044, Figure 3C), indicating the link of *KMT2D* mutations with NOTCH signaling activation. Among genes associated with NOTCH signaling pathway, MYC is the most important downstream target gene 22. As revealed by RNA sequencing, *KMT2D*^mut^ patients presented with increased *MYC* and *NOTCH1* expression (p=0.004, Figure 3D and p=0.049, Figure 3E). MYC expression showed positive linear correlation with NOTCH1 expression (p=0.006, Supplementary Figure 4A).

Among genes associated with NOTCH signaling pathway (Figure 3F), FBXW7 is a critical NOTCH suppressor and negatively regulates NOTCH cascade through downregulation of NICD 11. As expected, compared with *KMT2D*^wt^ patients, FBXW7 expression decreased in* KMT2D*^mut^ patients (p=0.043, Supplementary Figure 4B). By quantitative RT-PCR, *KMT2D*^R5432Q^ and *KMT2D*^kd^ resulted in significantly decreased expression of FBXW7 in SU-DHL-4 and U-2932 cells (Supplementary Figure 4C). ChIP assay showed lower occupancies of H3K4me3 in the proximal promoter areas of *FBXW7* in *KMT2D*^R5432Q^ and *KMT2D*^kd^ SU-DHL-4 cells than those in *KMT2D*^wt^ and scramble SU-DHL-4 cells (Figure 3G). In consistent with ChIP assay, FBXW7 were downregulated, while NICD and MYC were upregulated in *KMT2D*^C5092R^, *KMT2D*^G5182fs^, *KMT2D*^W5395X^, *KMT2D*^R5432Q^ and *KMT2D*^kd^ cells by western blot (Figure 3H). Besides, we used NOTCH inhibitor (γ-secretase inhibitor, GSI-I) to treat SU-DHL-4 cells. As a consequence of NICD downregulation by GSI-I, MYC expression was reduced in *KMT2D*^R5432Q^ and *KMT2D*^kd^ SU-DHL-4 cells (Figure 3I). Together, these data suggested that *KMT2D* mutations downregulated *FBXW7* and activated NOTCH/MYC signaling.

### *KMT2D* mutations promoted tumor-induced Treg cell trafficking via upregulation of TGF-β1

**Figure 4:**
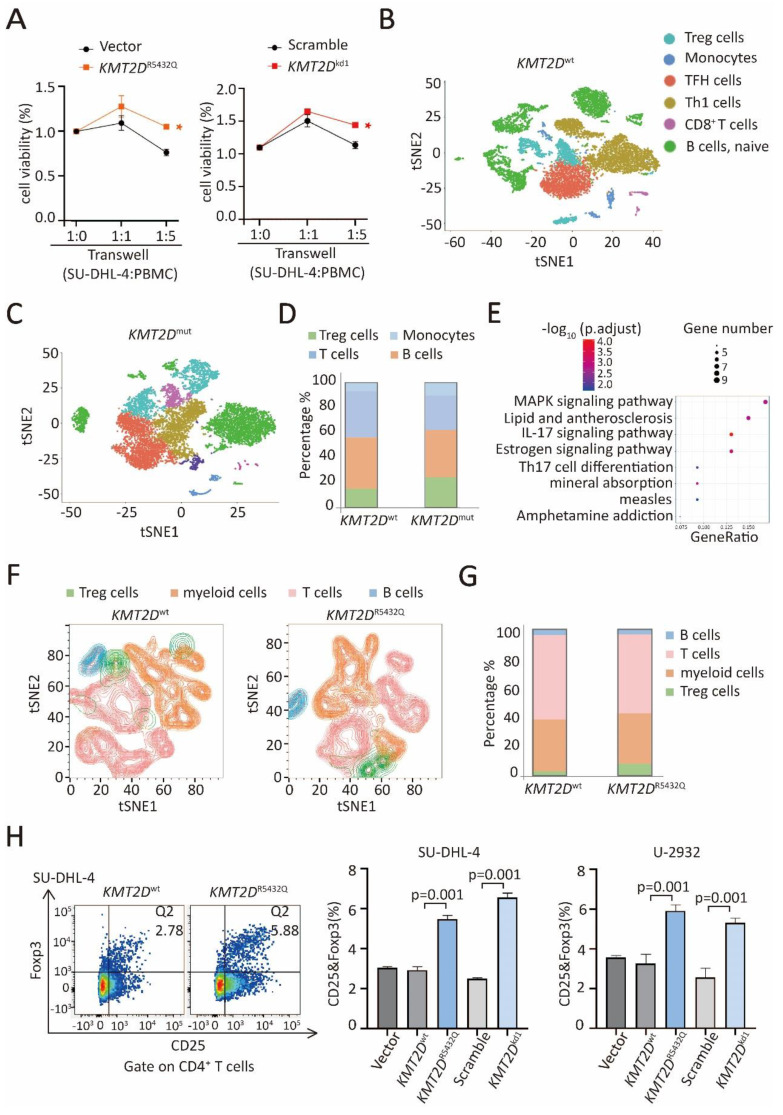
*** KMT2D* mutations promoted Treg cell trafficking.** (**A**). The viability of *KMT2D*^R5432Q^ SU-DHL-4 cells (left panel) and *KMT2D*^kd^ (right panel) when co-cultured with peripheral blood mononuclear cells (PBMCs) at 1:1 ratio or 1:5 ratio for 72 hours. *p < 0.05 comparing with Vector or Scramble SU-DHL-4 cells. (**B, C**). Main sub-clones shown by t-distributed stochastic neighbor embedding (tSNE) mapping of *KMT2D*^wt^ and *KMT2D*^mut^ DLBCL patients. Fractions of relative proportions of main sub-clones. (**D**). Fractions of relative proportions of sub-clones. (**E**). Pathway enrichment analysis of Treg cells in* KMT2D*^mut^ or *KMT2D*^wt^ patients according to KEGG database. (**F**). Main sub-clones shown by tSNE mapping of *KMT2D*^wt^, *KMT2D*^R5432Q^ SU-DHL-4 cells, co-cultured with PBMCs of 1:5 ratio for 72h. (**G**). Fractions of relative proportions of PBMC sub-clones. (**H**). Flow cytometry analysis of Treg cells markers (CD25 and Foxp3) in PBMCs, co-cultured with *KMT2D*^wt^, *KMT2D*^R5432Q^, Scramble, *KMT2D*^kd^ SU-DHL-4 and U-2932 cells for 72h.

*KMT2D*^R5432Q^ and *KMT2D*^kd^ induced a significantly increase in cell growth when co-cultured with peripheral blood mononuclear cells (PBMCs), as compared to Vector or Scramble cells, indicating that proliferation of B-lymphoma cells was influenced by the tumor microenvironment (Figure 4A). In order to explore the tumor microenvironmental change in *KMT2D*^mut^ patients, we performed single-cell RNA sequencing on 6 DLBCL patients, including 3 *KMT2D*^mut^ patients and 3 *KMT2D*^wt^ patients. Different cluster represented Treg cells, as well as B cells, T cells (non-Treg cells), and monocytes (Figure 4B and 4C). The number of Treg cells was increased in *KMT2D*^mut^ patients, as compared to *KMT2D*^wt^ patients. No difference was observed in other type of cells (Figure 4D).

KEGG pathway enrichment of differently expressed gene of Treg cells in *KMT2D*^mut^ patients revealed significant MAPK pathway activation (Figure 4E). Given that Treg cells were significantly increased in *KMT2D*^mut^ patients by RNA sequencing and single-cell sequencing, we performed multiple color flowcytometry analysis in SU-DHL-4 cells co-cultured with PBMCs, different clusters represented Treg cells, B cells, T cells (excluding Treg cells) and myeloid cells (Figure 4F). In the co-culture system, the number of CD4^+^Foxp3^+^ Treg cells was increased in *KMT2D*^R5432Q^ SU-DHL-4 cells, as compared to *KMT2D*^wt^ cells, without difference observed in other type of cells (Figure 4G). Next, we performed flow cytometry analysis in SU-DHL-4 and U-2932 cells, *KMT2D*^R5432Q^ and *KMT2D*^kd^ induced a significantly high level of CD25^+^Foxp3^+^ Treg cells than *KMT2D*^wt^ or Scramble cells, when co-cultured with PBMCs (Figure 4H).

**Figure 5:**
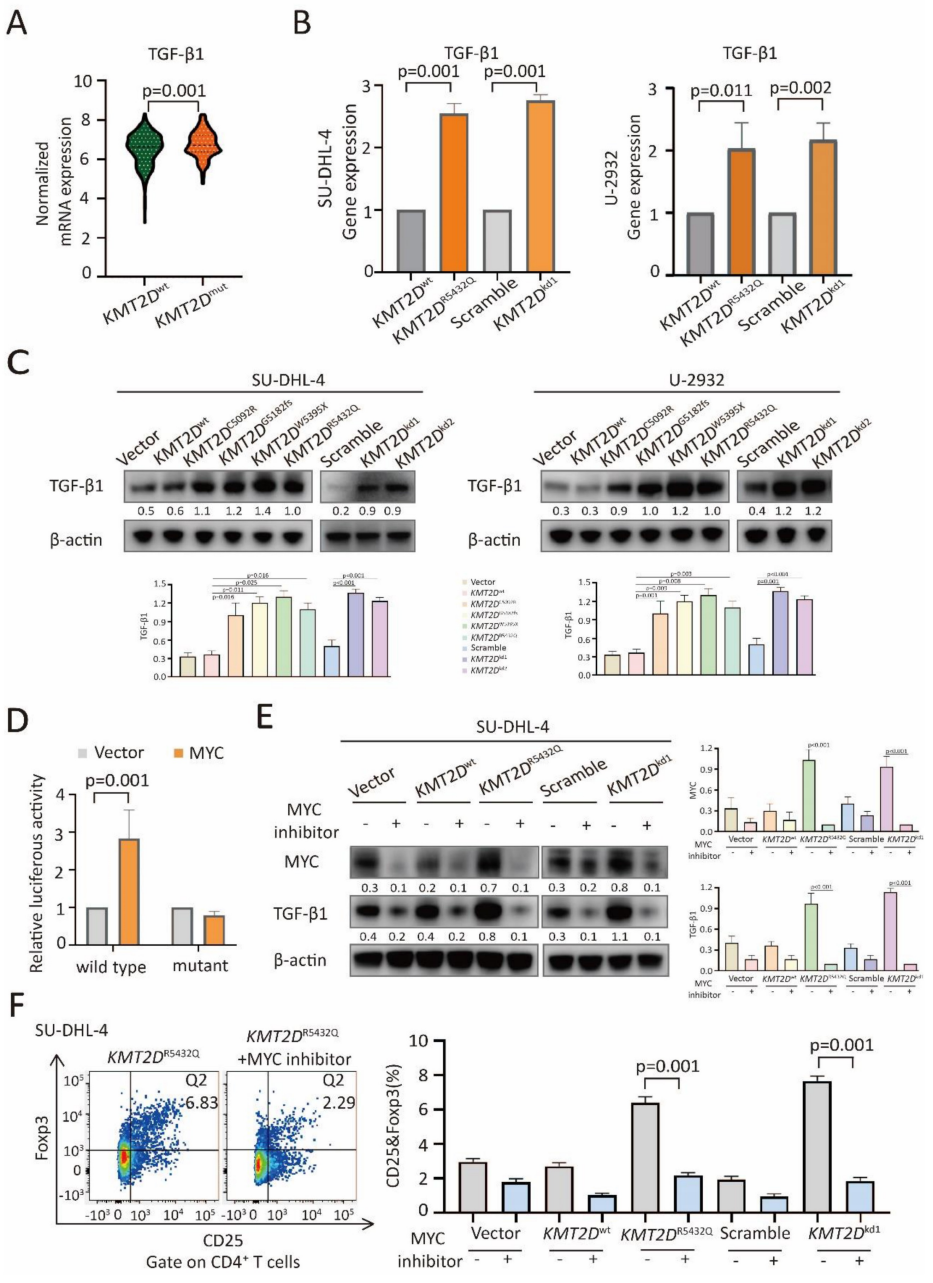
*** KMT2D* mutations promoted Treg cell trafficking via TGF-β1 axis.** (**A**). Normalized mRNA expression of TGF-β1 in tumor samples of DLBCL patients with or without *KMT2D* mutations as revealed by RNA sequencing data. (**B**). Relative gene expression of TGF-β1 in *KMT2D*^R5432Q^ and *KMT2D*^kd^ SU-DHL-4 and U-2932 cells, as compared to *KMT2D*^wt^ or Scramble cells by quantitative real-time PCR (RT-PCR). (**C**). Protein expression of TGF-β1 in *KMT2D*^C5092R^, *KMT2D*^G5182fs^, *KMT2D*^W5395X^, *KMT2D*^R5432Q^, *KMT2D*^kd1^ and *KMT2D*^kd2^ SU-DHL-4 and U-2932 cells, as compared to *KMT2D*^wt^ or Scramble cells by western blot. β-actin was used as a loading control. (**D**). Luciferase reporter assay in HEK-293T cells indicated the luciferase activity after the co-transfection with c-Myc wild-type and mutant. (**E**). Protein expression of MYC and TGF-β1 in *KMT2D*^R5432Q^ and *KMT2D*^kd^ SU-DHL-4 cells by western blot with and without MYC inhibitor (50μm) treatment for 48h. β-actin was used as a loading control. (**F**). Flow cytometry analysis of Treg cells markers (CD25 and Foxp3) in PBMCs, co-cultured with *KMT2D*^R5432Q^ or *KMT2D*^kd^ SU-DHL-4 cells with or without MYC inhibitor treatment (50μm) for 72h.

TGF-β1 and IL-10 are essential in Treg cell trafficking in tumor microenvironment 12, 23. Comparing with *KMT2D*^wt^ patients, TGF-β1 expression was increased in* KMT2D*^mut^ patients (p=0.001, Figure 5A), while IL-10 remained unchanged (Supplementary Figure 5). By quantitative RT-PCR (Figure 5B), *KMT2D*^R5432Q^ and *KMT2D*^kd^ resulted in significantly increased TGF-β1 expression at transcriptional level.

In consistent with RT-PCR assay, TGF-β1 was upregulated in *KMT2D*^C5092R^, *KMT2D*^G5182fs^, *KMT2D*^W5395X^, *KMT2D*^R5432Q^ and *KMT2D*^kd^ cells at protein level by western blot (Figure 5C). The transcription factor MYC can bind with the promoter region of TGF-β1 24, 25. As revealed by luciferase reporter assay, MYC positively regulated the transcriptional activity of the TGF-β1 promoter region (-133 to -127) 24 in HEK-293T cells (Figure 5D), suggesting that MYC targeted TGF-β1 through this binding site. To determine the role of NOTCH/MYC signaling in TGF-β1 upregulation and Treg cell trafficking, we used MYC inhibitor to treat SU-DHL-4 cells. As a consequence of MYC downregulation by MYC inhibitor, TGF-β1 expression was reduced in both *KMT2D*^R5432Q^ and *KMT2D*^kd^ cells by western blot (Figure 5E). Correspondingly, Treg cells were decreased upon MYC inhibitor treatment by flow cytometry analysis (Figure 5F). Together, our data demonstrated that *KMT2D* mutations induced NOTCH-MYC activation, as well as MYC-dependent TGF-β1 secretion, and resulted in tumor induced-Treg cell trafficking.

### *KMT2D* mutations promoted tumor-induced Treg cell trafficking via FBXW7-NOTCH-MYC/TGF-β1 axis in PBMC-transferred NOD-scid murine models

**Figure 6:**
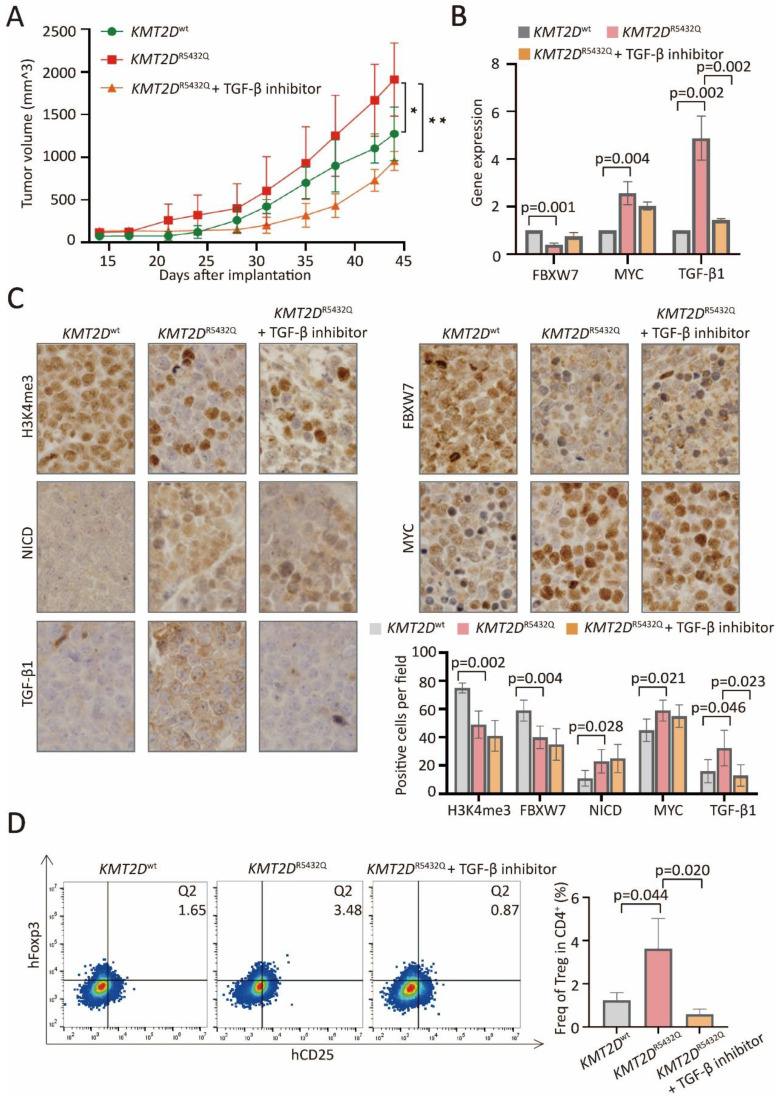
*** KMT2D* mutations promoted tumor-induced Treg cell trafficking via FBXW7-NOTCH/MYC/TGF-β1 axis in PBMC-transferred M-NSG murine models.** (**A**). Tumor volume of murine models injected subcutaneously with *KMT2D*^R5432Q^ SU-DHL-4 cells. Error bars represent SD (n = 5). *p < 0.05; **p < 0.01; comparing with those of *KMT2D*^wt^ SU-DHL-4 cells. (**B**). RT-PCR of FBXW7, MYC and TGF-β1 in *KMT2D*^wt^, *KMT2D*^R5432Q^ and TGF-β inhibitor-treated *KMT2D*^R5432Q^ murine models. (**C**). Immunohistochemical assay of H3K4me3, FBXW7, NICD, MYC and TGF-β1 in *KMT2D*^wt^, *KMT2D*^R5432Q^ and TGF-β inhibitor-treated *KMT2D*^R5432Q^ murine models. (**D**). Flow cytometry analysis of Treg markers (CD25 and Foxp3) in *KMT2D*^wt^, untreated *KMT2D*^R5432Q^ and TGF-β inhibitor-treated *KMT2D*^R5432Q^ murine models.

In PBMC-transferred M-NSG murine models established with subcutaneous injection of SU-DHL-4 cells, the tumor size formed in mice with *KMT2D*^R5432Q^ were significantly increased, as compared to *KMT2D*^wt^, which could be counteracted by TGF-β inhibitor (Figure 6A). Downregulated expression of FBXW7, upregulated expression of MYC and TGF-β1 were detected in *KMT2D*^R5432Q^ murine models by quantitative RT-PCR (Figure 6B). Downregulated protein expression of H3K4me3 and FBXW7, upregulated expression of NICD, MYC and TGF-β1 were detected in *KMT2D*^R5432Q^ murine models by immunohistochemistry (Figure 6C). Upregulation of TGF-β1 could be reversed by TGF-β inhibitor. Flow cytometry analysis showed increased amounts of tumor-induced Treg cells in *KMT2D*^R5432Q^ murine models, which could also be interacted by TGF-β inhibitor (Figure 6D).

## Discussion

*KMT2D* mutations were highly recurrent in DLBCL and significantly associated with poor clinical outcomes. Here we provided further clinical evidence that dysregulation of histone methylation was important tumorigenic event in DLBCL. Functionally, *KMT2D* mutations occur early during tumorigenesis in DLBCL by inhibiting global H3K4 methylation, perturbing the expression of tumor suppressor genes that control B-cell activating pathways 4, 26. In B-lymphoma cells and in tumor samples of DLBCL patients, we showed that *KMT2D* mutations decreased H3K4me3, downregulated tumor suppressor gene *FBXW7* 27, and more importantly, activated downstream NOTCH/MYC signaling, which are critically involved in DLBCL 28. Indeed, KMT2D contains multiple N-terminal phosphodegrons required to promote interaction with FBXW7, which could control proteasome-mediated degradation of oncoproteins particularly as NOTCH 27, 29. As downstream effectors of NOTCH signaling pathway 30, 31, MYC was correspondingly upregulated in a NOTCH-dependent manner. As similar mechanism of action, loss of function of *KMT2D* inhibits tumor suppressor genes *DNMT3A* and *BCL6*, upregulates NOTCH pathway, and induces medulloblastoma in murine models 6.

During T-cell lymphomagenesis, *KMT2D* mutations were also identified as a prognostic marker through altering the stability of the activator complex, affecting the expression of tumor suppressor genes and NOTCH signaling pathway 32. Together, *KMT2D* mutations promoted tumor progression by regulating FBXW7-NOTCH-MYC axis in DLBCL.

*KMT2D* deficiency affects expression of a specific set of genes significantly associated with immune signaling pathways, including Treg cell differentiation 26, 33. Recent study has shown that *KMT2D*^mut^ cells exhibit increased protein turnover and IFN-γ-stimulated antigen presentation across multiple cancer types. *KMT2D*^mut^ liver cancer is characterized by increased immune infiltration such as CD45+ immune cells, CD4+ T cells, and macrophages 34. We revealed, both *in vitro* and *in vivo*, that *KMT2D* mutations induced B-lymphoma cell proliferation, with the presence of tumor microenvironment. Single-cell RNA sequencing further confirmed that *KMT2D* mutations increased Treg cell trafficking in DLBCL. Tumor-induced Treg cells 14 contributed to tumor progression by expressing not only a plethora of genes with well-established immune suppressive function, but also genes that support a committed Foxp3+ Treg phenotype 35. To our knowledge, this is the first report on the impact of *KMT2D* mutations on Treg cells, suggesting an alternative mechanism of epigenetic alterations on tumor microenvironment in DLBCL.

Treg cells within tumor microenvironment could suppress immunosurveillance function to promote lymphoma progression 36, 37. The NOTCH signaling pathway and downstream effector MYC are critically involved in Treg cell recruiting activity and function 38, 39. Experimentally, MYC could accelerate TGF-β1 transcription and promote oral squamous cell carcinoma tumorigenesis 24. TGF-β1 is a potent immunosuppressive cytokine and stimulates the differentiation of Treg cells 40, 41. TGF-β receptor inhibitor SB431542 significantly reduced Treg cells and the tumor burden in fibrosarcoma-bearing mice 42. Here we found that tumor cell growth and tumor-induced Treg cell trafficking were increased in *KMT2D*^mut^ DLBCL, which could be subsequently counteracted by TGF-β inhibitor. Our data demonstrated the NOTCH-MYC/TGF-β1 axis as an important mechanism of tumor progression related to tumor-induced Treg cells in DLBCL. Considering that the NOTCH inhibitor has an off-target effect and is toxic if applied systemically, designing a more effective way to target TGF-β1 can be a feasible immunomodulatory intervention for DLBCL treatment.

## Conclusions

In conclusion, mutations of histone methylation gene *KMT2D* may modulate tumor-induced Treg cell trafficking via the FBXW7-NOTCH-MYC/TGF-β1 axis. Aberrant histone methylation on tumor microenvironment can be considered as an alternative mechanism of tumor progression in DLBCL.

## Supplementary Material

Supplementary figures and tables.

## Figures and Tables

**Table 1:** Clinical characteristics of the patients with DLBCL (n=761)

Characteristics	*KMT2D*^wt^ (n=618)		*KMT2D*^mut^ (n=143)	*p* value
Gender				
Male	378 (45.0%)	64 (44.8%)		0.961
Female	340 (55.0%)	79 (55.2%)		
Age				
≤ 60	349 (56.5%)	92 (64.3%)		0.211
> 60	268 (43.4%)	51 (35.7%)		
ECOG score				
≤ 1	565 (91.4%)	123 (86.6%)		0.078
>1	53 (8.6%)	19 (13.4%)		
Ann Arbor stage				
I-II	349 (56.5%)	62 (43.4%)		0.003
III-IV	269 (43.5%)	80 (55.9%)		
Serum LDH				
Normal	328 (53.2%)	71 (49.7%)		0.449
Elevated	289 (46.8%)	72 (50.3%)		
IPI				
0-2	437 (70.7%)	92 (64.3%)		0.136
3-5	181 (29.3%)	51 (35.7%)		
Cell of origin (Hans)				
GCB	241 (39.0%)	56 (39.2%)		0.997
Non-GCB	373 (60.4%)	86 (60.1%)		
Extranodal involvement				0.871
≤ 1	447 (72.6%)	104 (73.2%)		
> 1	169 (27.4%)	38 (26.8%)		
MYC Expression				
< 40%	347 (67.0%)	71 (55.5%)		0.015
≥ 40%	171 (33.0%)	57 (44.5%)		
BCL2 Expression				
< 50%	233 (42.2%)	48 (36.1%)		0.198
≥ 50%	319 (57.8%)	85 (63.9%)		
				

## Abbreviations

ChIP: Chromatin immunoprecipitation
DLBCL: diffuse large B-cell lymphoma
GSEA: Gene Set Enrichment Analysis
KEGG: Kyoto Encyclopedia of Genes and Genomes
NICD: intracellular portions of NOTCH1
OS: overall survival
PFS: progression-free survival
PBMC: peripheral blood mononuclear cell
RT-PCR: quantitative real-time PCR
Treg cells: regulatory T cells
tSNE: t-distributed stochastic neighbor embedding
WGS/WES: whole-genome/exome sequencing
