# Supplementary material for: KMT2D mutations promoted tumor progression in diffuse large B-cell lymphoma through altering tumor-induced regulatory T cell trafficking via FBXW7-NOTCH-MYC/TGF-β1 axis
Source: Int J Biol Sci. 2024 Jul 15;20(10):3972–85. doi: 10.7150/ijbs.93349 (PMC11302885; doi:10.7150/ijbs.93349)
Supplement: Supplementary file 1 — Supplementary figures and tables. [file ijbsv20p3972s1.pdf]

Supplementary Figure 1 Forest plot of univariate analysis on progression-free survival of selected subgroups

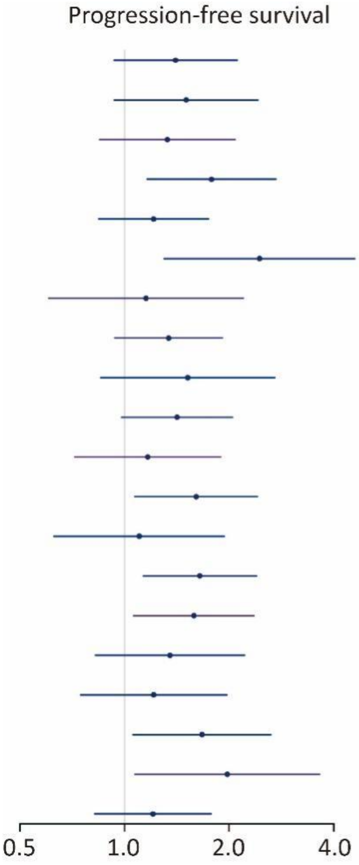

	HR (95%CI)	P value
Male	1.403 (0.933-2.110)	0.115
Female	1.504 (0.934-2.423)	0.106
Age ≤ 60	1.328 (0.846-2.084)	0.229
Age > 60	1.780 (1.160-2.731)	0.012
ECOG score ≤ 1	1.212 (0.842-1.745)	0.311
ECOG score > 1	2.447 (1.300-4.607)	0.008
Ann Arbor stage I-II	1.153 (0.605-2.197)	0.671
Ann Arbor stage III-IV	1.338 (0.937-1.912)	0.118
Normal serum LDH	1.520 (0.853-2.708)	0.172
Elevated serum LDH	1.416 (0.980-2.046)	0.073
IPI 0-2	1.166 (0.717-1.900)	0.542
IPI 3-5	1.608 (1.070-2.415)	0.028
GCB	1.102 (0.626-1.940)	0.739
Non-GCB	1.647 (1.131-2.400)	0.012
Extranodal involvement ≤ 1	1.584 (1.062-2.361)	0.030
Extranodal involvement > 1	1.352 (0.824-2.220)	0.246
MYC expression < 40%	1.213 (0.746-1.971)	0.446
MYC expression ≥ 40%	1.672 (1.058-2.641)	0.033
BCL2 expression < 50%	1.978 (1.071-3.652)	0.040
BCL2 expression ≥ 50%	1.207 (0.819-1.778)	0.350

Supplementary Figure 2 *KMT2D* mutations induced Treg cell activation by Gene Set Enrichment Analysis (GSEA)

Prediction of immune subpopulations normalized enrichment scores for DLBCL patients with *KMT2D* mutations using GSEA. GSEA showing association of Treg cells gene signatures with *KMT2D* mutations in diffuse large B-cell lymphoma (DLBCL) patients. Enrichment scores were listed with p-value. NES, normalized enrichment score; FDR, false discovery rate.

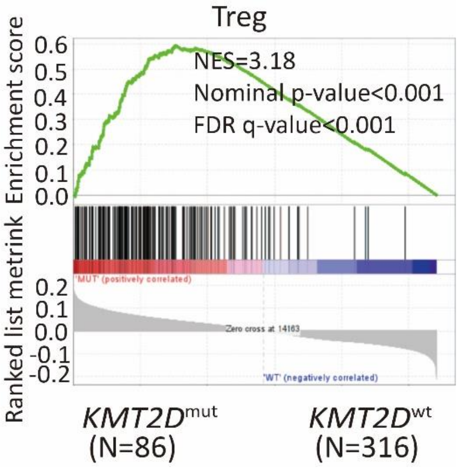

Supplementary Figure 3 Schematic representation of *KMT2D* mutations identified in patients

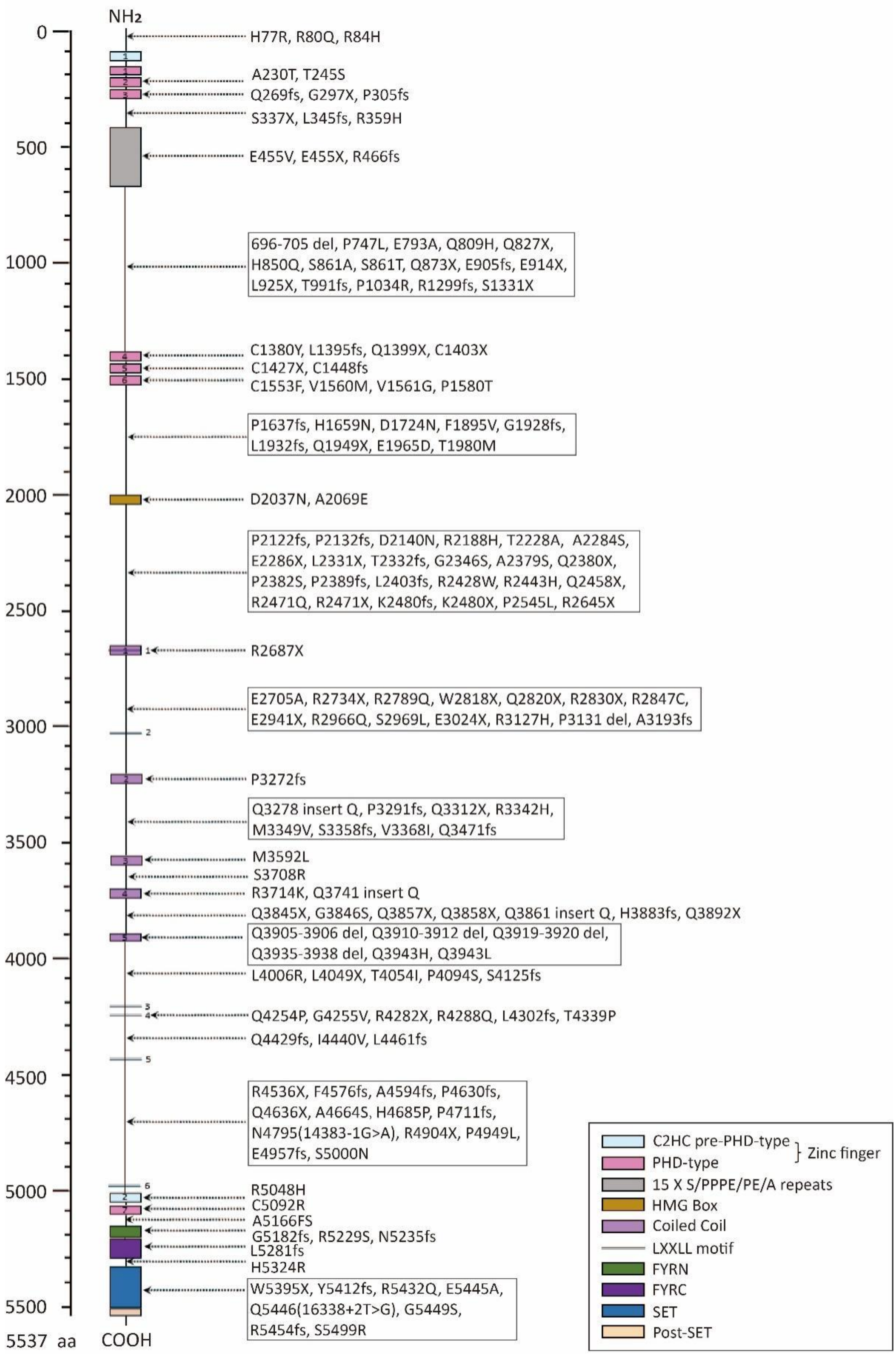

Supplementary Figure 4 *KMT2D* mutations inhibited FBXW7 expression

(**A**). Correlations between MYC and NOTCH1 according to Normalized mRNA expression as revealed by RNA sequencing.

(**B**). Normalized mRNA expression of FBXW7 in tumor samples of DLBCL patients with or without *KMT2D* mutations as revealed by RNA sequencing.

(**C**). Relative gene expression of FBXW7 in *KMT2D*^{R5432Q} or *KMT2D*^{kd} SU-DHL-4 and U-2932 cells, as compared to *KMT2D*^{wt} or Scramble cells by quantitative real-time PCR.

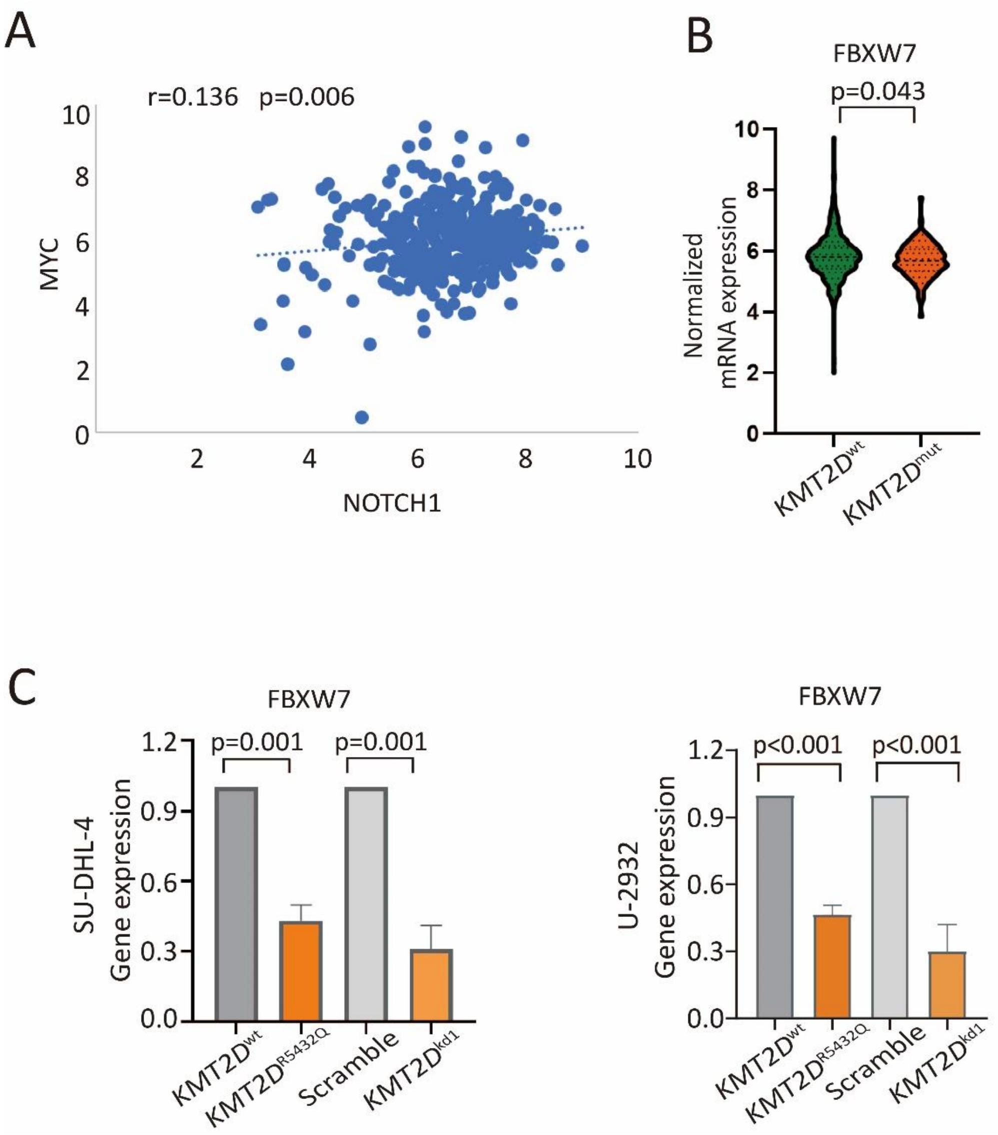

Supplementary Figure 5 Normalized mRNA expression of IL10 in tumor samples of DLBCL patients with or without *KMT2D* mutations as revealed by RNA sequencing

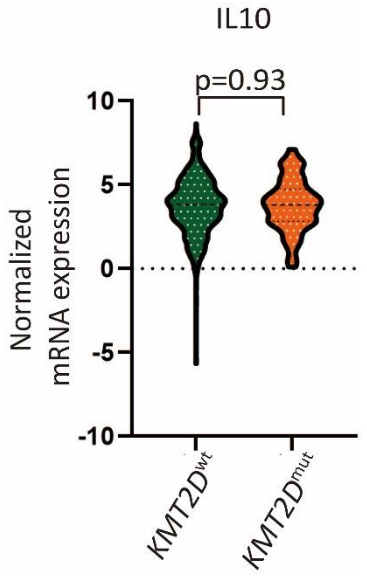

Supplementary Table 1. Sequences of shRNA for KMT2D.

shRNA1	TCGCCTCAAGAAATGGAAA
shRNA2	TCTACATGTTCCGAATAAA

Supplementary Table 2. Sequences of primers for RT-PCR.

Name	Forward	Reverse
TGF-β1	CAGCAACAATTCCTGGCGATAC	TCAACCACTGCCGCACAACT
FBXW7	GGCGCCGCGGCTCTTTTCTA	GCTGCCCACAGAGAGCAGTTCC

Supplementary Table 3. *KMT2D* mutations identified in 761 DLBCL patients.

Patient ID	Chr	Start	End	Ref	Alt	ExonicFunc.refGene	AAChange.refGene
1	chr12	49416416	49416416	C	T	nonsynonymous SNV	KMT2D:NM_003482:exon51:c.G16295A:p.R5432Q
2	chr12	49433620	49433620	G	A	stopgain	KMT2D:NM_003482:exon31:c.C7933T:p.R2645X
3	chr12	49425584	49425584	G	-	frameshift deletion	KMT2D:NM_003482:exon39:c.12904delC:p.L4302fs
4	chr12	49446774	49446777	AGAG	-	frameshift deletion	KMT2D:NM_003482:exon8:c.1033_1036del:p.L345fs
5	chr12	49425625	49425625	C	T	nonsynonymous SNV	KMT2D:NM_003482:exon39:c.G12863A:p.R4288Q
6	chr12	49444388	49444400	GCTCAGGGTCAGT	-	frameshift deletion	KMT2D:NM_003482:exon11:c.2971_2983del:p.T991fs
7	chr12	49426327	49426327	G	A	nonsynonymous SNV	KMT2D:NM_003482:exon39:c.C12161T:p.T4054I
8	chr12	49424442	49424442	-	C	frameshift insertion	KMT2D:NM_003482:exon41:c.13780dupG:p.A4594fs
9	chr12	49432242	49432242	C	T	nonsynonymous SNV	KMT2D:NM_003482:exon34:c.G8897A:p.R2966Q
10	chr12	49420475	49420475	A	G	nonsynonymous SNV	KMT2D:NM_003482:exon48:c.T15274C:p.C5092R
11	chr12	49439706	49439706	G	T	nonsynonymous SNV	KMT2D:NM_003482:exon18:c.C4738A:p.P1580T
12	chr12	49426730	49426732	GCT	-	nonframeshift deletion	KMT2D:NM_003482:exon39:c.11756_11758del:p.3919_3920del
13	chr12	49425170	49425170	T	C	nonsynonymous SNV	KMT2D:NM_003482:exon39:c.A13318G:p.I4440V
14	chr12	49445039	49445039	C	G	nonsynonymous SNV	NM_003482:exon10:p.Q809H
15	chr12	49431305	49431305	-	TGC	nonframeshift insertion	KMT2D:NM_003482:exon34:c.9833_9834insGCA:p.Q3278delinsQQ
16	chr12	49435135	49435135	C	T	nonsynonymous SNV	KMT2D:NM_003482:exon31:c.G6418A:p.D2140N
17	chr12	49433919	49433919	G	A	nonsynonymous SNV	KMT2D:NM_003482:exon31:c.C7634T:p.P2545L
18	chr12	49441788	49441800	TGCGAACAGGCAA	-	frameshift deletion	KMT2D:NM_003482:exon14:c.4184_4196del:p.L1395fs
19	chr12	49433388	49433388	G	A	stopgain	KMT2D:NM_003482:exon32:c.C8059T:p.R2687X
20	chr12	49432069	49432069	C	A	stopgain	KMT2D:NM_003482:exon34:c.G9070T:p.E3024X
21	chr12	49436381	49436417	AGGAGTCCATTGGGCTGCTGGAGGGCAGATTGCCCAA	-	frameshift deletion	KMT2D:NM_003482:exon27:c.5794_5830del:p.L1932fs
22	chr12	49440465	49440466	CA	-	frameshift deletion	KMT2D:NM_003482:exon15:c.4344_4345del:p.C1448fs
23	chr12	49435157	49435157	G	-	frameshift deletion	KMT2D:NM_003482:exon31:c.6396delC:p.P2132fs
24	chr12	49434703	49434703	C	A	nonsynonymous SNV	KMT2D:NM_003482:exon31:c.G6850T:p.A2284S
25	chr12	49424741	49424741	G	A	stopgain	KMT2D:NM_003482:exon40:c.C13606T:p.R4536X
26	chr12	49442916	49442916	G	C	stopgain	KMT2D:NM_003482:exon12:c.C3992G:p.S1331X
27	chr12	49434418	49434418	C	A	nonsynonymous SNV	KMT2D:NM_003482:exon31:c.G7135T:p.A2379S
28	chr12	49434703	49434703	C	A	nonsynonymous SNV	KMT2D:NM_003482:exon31:c.G6850T:p.A2284S
29	chr12	49446734	49446734	C	T	nonsynonymous SNV	KMT2D:NM_003482:exon8:c.G1076A:p.R359H
30	chr12	49434409	49434409	G	A	nonsynonymous SNV	NM_003482:exon31:p.P2382S
31	chr12	49434990	49434990	C	T	nonsynonymous SNV	KMT2D:NM_003482:exon31:c.G6563A:p.R2188H
32	chr12	49438294	49438294	G	T	nonsynonymous SNV	KMT2D:NM_003482:exon20:c.C4975A:p.H1659N

33	chr12	49426952	49426952	C	T	nonsynonymous SNV	NM_003482:exon39:p.G3846S
34	chr12	49416115	49416115	G	-	frameshift deletion	KMT2D:NM_003482:exon52:c.16360delC:p.R5454fs
35	chr12	49434181	49434181	G	A	stopgain	KMT2D:NM_003482:exon31:c.C7372T:p.Q2458X
36	chr12	49447286	49447293	GGGCACTG	-	frameshift deletion	KMT2D:NM_003482:exon6:c.805_812del:p.Q269fs
37	chr12	49447031	49447031	-	T	frameshift insertion	KMT2D:NM_003482:exon7:c.912dupA:p.P305fs
38	chr12	49426772	49426774	GCT	-	nonframeshift deletion	KMT2D:NM_003482:exon39:c.11714_11716del:p.3905_3906del
39	chr12	49445352	49445378	GAGTCCTCAGGTGGTGGGGATGTGGGG	-	nonframeshift deletion	KMT2D:NM_003482:exon10:c.2088_2114del:p.696_705del
40	chr12	49423205	49423205	T	G	nonsynonymous SNV	NM_003482:exon43:p.H4685P
41	chr12	49427364	49427364	G	C	nonsynonymous SNV	KMT2D:NM_003482:exon39:c.C11124G:p.S3708R
42	chr12	49431114	49431114	C	T	nonsynonymous SNV	KMT2D:NM_003482:exon34:c.G10025A:p.R3342H
43	chr12	49448481	49448481	T	C	nonsynonymous SNV	KMT2D:NM_003482:exon3:c.A230G:p.H77R
44	chr12	49431542	49431560	GCTGGGGGTCAGCAGGTGA	-	frameshift deletion	KMT2D:NM_003482:exon34:c.9579_9597del:p.A3193fs
45	chr12	49420251	49420251	A	-	frameshift deletion	KMT2D:NM_003482:exon48:c.15498delT:p.A5166fs
46	chr12	49424156	49424156	G	A	stopgain	KMT2D:NM_003482:exon42:c.C13906T:p.Q4636X
47	chr12	49434490	49434559	CAGGGGGTGGCTCCTGGGGCCTTAGGCCCAAGCCCGGGCTCTGGGGCTCTACCTGAGATGCCCGAGGGGT	-	frameshift deletion	KMT2D:NM_003482:exon31:c.6994_7063del:p.T2332fs
48	chr12	49447410	49447410	C	T	nonsynonymous SNV	KMT2D:NM_003482:exon6:c.G688A:p.A230T
49	chr12	49434871	49434871	T	C	nonsynonymous SNV	KMT2D:NM_003482:exon31:c.A6682G:p.T2228A
50	chr12	49426814	49426814	G	A	stopgain	NM_003482:exon39:p.Q3892X
51	chr12	49434142	49434142	G	A	stopgain	KMT2D:NM_003482:exon31:c.C7411T:p.R2471X
52	chr12	49416377	49416377	T	G	nonsynonymous SNV	NM_003482:exon51:p.E5445A
53	chr12	49420204	49420204	C	-	frameshift deletion	KMT2D:NM_003482:exon48:c.15545delG:p.G5182fs
54	chr12	49434225	49434225	C	T	nonsynonymous SNV	KMT2D:NM_003482:exon31:c.G7328A:p.R2443H
55	chr12	49439859	49439859	A	C	nonsynonymous SNV	NM_003482:exon17:p.V1561G
56	chr12	49441789	49441789	G	A	stopgain	KMT2D:NM_003482:exon14:c.C4195T:p.Q1399X
57	chr12	49432318	49432318	C	A	stopgain	KMT2D:NM_003482:exon34:c.G8821T:p.E2941X
58	chr12	49434409	49434409	G	A	nonsynonymous SNV	NM_003482:exon31:p.P2382S
59	chr12	49425099	49425106	CAAGAGCA	-	frameshift deletion	KMT2D:NM_003482:exon39:c.13382_13389del:p.L4461fs
60	chr12	49427265	49427265	-	TGC	nonframeshift insertion	KMT2D:NM_003482:exon39:c.11222_11223insGCA:p.Q3741delinsQQ
61	chr12	49438580	49438580	G	-	frameshift deletion	KMT2D:NM_003482:exon19:c.4910delC:p.P1637fs
62	chr12	49444987	49444987	G	A	stopgain	KMT2D:NM_003482:exon10:c.C2479T:p.Q827X
63	chr12	49426955	49426955	G	A	stopgain	KMT2D:NM_003482:exon39:c.C11533T:p.Q3845X

64	chr12	49447410	49447410	C	T	nonsynonymous SNV	
65	chr12	49424173	49424173	G	-	frameshift deletion	
66	chr12	49420064	49420064	G	T	nonsynonymous SNV	KMT2D:NM_003482:exon48:c.C15685A:p.R5229S
67	chr12	49434141	49434141	C	T	nonsynonymous SNV	KMT2D:NM_003482:exon31:c.G7412A:p.R2471Q
68	chr12	49426115	49426115	-	AG	frameshift insertion	KMT2D:NM_003482:exon39:c.12372_12373insCT:p.S4125fs
69	chr12	49426838	49426839	TG	-	frameshift deletion	KMT2D:NM_003482:exon39:c.11649_11650del:p.H3883fs
70	chr12	49416526	49416526	C	T	stopgain	KMT2D:NM_003482:exon51:c.G16185A:p.W5395X
71	chr12	49444692	49444692	A	T	stopgain	KMT2D:NM_003482:exon10:c.T2774A:p.L925X
72	chr12	49431324	49431324	G	-	frameshift deletion	KMT2D:NM_003482:exon34:c.9815delC:p.P3272fs
73	chr12	49439859	49439859	A	C	nonsynonymous SNV	KMT2D:NM_003482:exon17:c.T4682G:p.V1561G
74	chr12	49436427	49436427	A	-	frameshift deletion	KMT2D:NM_003482:exon27:c.5784delT:p.G1928fs
75	chr12	49424442	49424442	-	C	frameshift insertion	KMT2D:NM_003482:exon41:c.13780dupG:p.A4594fs
76	chr12	49426754	49426759	GTTGCT	-	nonframeshift deletion	KMT2D:NM_003482:exon39:c.11729_11734del:p.3910_3912del
77	chr12	49431746	49431748	AGG	-	nonframeshift deletion	KMT2D:NM_003482:exon34:c.9391_9393del:p.3131_3131del
78	chr12	49440529	49440529	A	T	stopgain	KMT2D:NM_003482:exon15:c.T4281A:p.C1427X
79	chr12	49444885	49444885	A	T	nonsynonymous SNV	KMT2D:NM_003482:exon10:c.T2581A:p.S861T
80	chr12	49432686	49432686	C	T	stopgain	KMT2D:NM_003482:exon34:c.G8453A:p.W2818X
81	chr12	49438001	49438001	C	T	nonsynonymous SNV	KMT2D:NM_003482:exon21:c.G5170A:p.D1724N
82	chr12	49436623	49436623	A	C	nonsynonymous SNV	KMT2D:NM_003482:exon26:c.T5683G:p.F1895V
83	chr12	49420750	49420750	C	T	nonsynonymous SNV	KMT2D:NM_003482:exon48:c.G14999A:p.S5000N
84	chr12	49426905	49426905	-	TGC	nonframeshift insertion	KMT2D:NM_003482:exon39:c.11582_11583insGCA:p.Q3861delinsQQ
85	chr12	49427714	49427714	T	G	nonsynonymous SNV	KMT2D:NM_003482:exon39:c.A10774C:p.M3592L
86	chr12	49433247	49433247	G	A	stopgain	KMT2D:NM_003482:exon32:c.C8200T:p.R2734X
87	chr12	49433333	49433333	T	G	nonsynonymous SNV	KMT2D:NM_003482:exon32:c.A8114C:p.E2705A
88	chr12	49426342	49426342	A	T	stopgain	KMT2D:NM_003482:exon39:c.T12146A:p.L4049X
89	chr12	49431759	49431759	C	T	nonsynonymous SNV	KMT2D:NM_003482:exon34:c.G9380A:p.R3127H
90	chr12	49434409	49434409	G	A	nonsynonymous SNV	KMT2D:NM_003482:exon31:c.C7144T:p.P2382S
91	chr12	49443476	49443476	G	-	frameshift deletion	KMT2D:NM_003482:exon11:c.3895delC:p.R1299fs
92	chr12	49434409	49434409	G	A	nonsynonymous SNV	KMT2D:NM_003482:exon31:c.C7144T:p.P2382S
93	chr12	49432233	49432233	G	A	nonsynonymous SNV	KMT2D:NM_003482:exon34:c.C8906T:p.S2969L
94	chr12	49415852	49415852	T	G	nonsynonymous SNV	KMT2D:NM_003482:exon53:c.A16495C:p.S5499R
95	chr12	49434409	49434409	G	A	nonsynonymous SNV	KMT2D:NM_003482:exon31:c.C7144T:p.P2382S
96	chr12	49037254	49037254	C	T	nonsynonymous SNV	KMT2D:NM_003482:exon34:c.G10102A:p.V3368I
97	chr12	49434409	49434409	G	A	nonsynonymous SNV	KMT2D:NM_003482:exon31:c.C7144T:p.P2382S

98	chr12	49027120	49027120	G	A	nonsynonymous SNV	KMT2D:NM_003482:exon48:c.C14846T:p.P4949L
99	chr12	49033581	49033581	G	C	nonsynonymous SNV	KMT2D:NM_003482:exon39:c.C11124G:p.S3708R
100	chr12	49032971	49032976	GTTGCT	-	nonframeshift deletion	KMT2D:NM_003482:exon39:c.11729_11734del:p.3910_3912del
101	chr12	49047992	49047992	G	T	stopgain	KMT2D:NM_003482:exon14:c.C4209A:p.C1403X
102	chr12	49034609	49034609	-	TGCA	frameshift insertion	KMT2D:NM_003482:exon36:c.10412_10413insTGCA:p.Q3471fs
103	chr12	49042259	49042259	G	A	nonsynonymous SNV	KMT2D:NM_003482:exon28:c.C5939T:p.T1980M
104	chr12	49042583	49042583	G	A	stopgain	KMT2D:NM_003482:exon27:c.C5845T:p.Q1949X
105	chr12	49032971	49032976	GTTGCT	-	nonframeshift deletion	KMT2D:NM_003482:exon39:c.11729_11734del:p.3910_3912del rs398123709
106	chr12	49030289	49030289	C	A	nonsynonymous SNV	KMT2D:NM_003482:exon42:c.G13990T:p.A4664S
107	chr12	49040632	49040632	G	A	stopgain	KMT2D:NM_003482:exon31:c.C7138T:p.Q2380X
108	chr12	49028142	49028142	C	T	splicing	KMT2D:NM_003482:exon46:c.14383-1G>A
109	chr12	49040779	49040779	G	-	stopgain	KMT2D:NM_003482:exon31:c.6991delC:p.L2331X
110	chr12	49054677	49054677	C	T	nonsynonymous SNV	KMT2D:NM_003482:exon3:c.G251A:p.R84H
111	chr12	49432600	49432600	G	A	nonsynonymous SNV	KMT2D:NM_003482:exon34:c.C8539T:p.R2847C
112	chr12	49444747	49444753	GTGGTTC	-	frameshift deletion	KMT2D:NM_003482:exon10:c.2713_2719del:p.E905fs
113	chr12	49446070	49446070	-	T	frameshift insertion	NM_003482:exon10:p.R466fs
114	chr12	49432651	49432651	G	A	stopgain	NM_003482:exon34:p.R2830X
115	chr12	49434409	49434409	G	A	nonsynonymous SNV	NM_003482:exon31:p.P2382S
116	chr12	49445226	49445226	G	A	nonsynonymous SNV	KMT2D:NM_003482:exon10:c.C2240T:p.P747L
116	chr12	49426208	49426208	G	A	nonsynonymous SNV	KMT2D:NM_003482:exon39:c.C12280T:p.P4094S
116	chr12	49435187	49435187	G	-	frameshift deletion	KMT2D:NM_003482:exon31:c.6366delC:p.P2122fs
117	chr12	49416468	49416477	TGGCTGCATA	-	frameshift deletion	KMT2D:NM_003482:exon51:c.16234_16243del:p.Y5412fs
117	chr12	49441845	49441845	C	T	nonsynonymous SNV	KMT2D:NM_003482:exon14:c.G4139A:p.C1380Y
118	chr12	49421039	49421039	G	A	stopgain	KMT2D:NM_003482:exon48:c.C14710T:p.R4904X
118	chr12	49434386	49434386	-	GGTT	frameshift insertion	KMT2D:NM_003482:exon31:c.7166_7167insAACC:p.P2389fs
119	chr12	49427265	49427265	-	TGC	nonframeshift insertion	KMT2D:NM_003482:exon39:c.11222_11223insGCA:p.Q3741delinsQQ
119	chr12	49448472	49448472	C	T	nonsynonymous SNV	KMT2D:NM_003482:exon3:c.G239A:p.R80Q
120	chr12	49420044	49420044	G	-	frameshift deletion	KMT2D:NM_003482:exon48:c.15705delC:p.N5235fs
120	chr12	49434517	49434517	C	T	nonsynonymous SNV	KMT2D:NM_003482:exon31:c.G7036A:p.G2346S
121	chr12	49446102	49446102	T	A	nonsynonymous SNV	KMT2D:NM_003482:exon10:c.A1364T:p.E455V
121	chr12	49446103	49446103	C	A	stopgain	KMT2D:NM_003482:exon10:c.G1363T:p.E455X
122	chr12	49431094	49431094	T	C	nonsynonymous SNV	KMT2D:NM_003482:exon34:c.A10045G:p.M3349V

122	chr12	49445352	49445378	GAGTCCTCAGGTGGTGGGGATGTGGGG	-	nonframeshift deletion	KMT2D:NM_003482:exon10:c.2088_2114del:p.696_705del
123	chr12	49427347	49427347	C	T	nonsynonymous SNV	NM_003482:exon39:p.R3714K
123	chr12	49435466	49435466	G	T	nonsynonymous SNV	NM_003482:exon30:p.A2069E
124	chr12	49434409	49434409	G	A	nonsynonymous SNV	NM_003482:exon31:p.P2382S
124	chr12	49447364	49447364	G	C	nonsynonymous SNV	NM_003482:exon6:p.T245S
125	chr12	49426919	49426919	G	A	stopgain	KMT2D:NM_003482:exon39:c.C11569T:p.Q3857X
125	chr12	49434271	49434271	G	A	nonsynonymous SNV	KMT2D:NM_003482:exon31:c.C7282T:p.R2428W
125	chr12	49425473	49425473	T	G	nonsynonymous SNV	NM_003482:exon39:p.T4339P
125	chr12	49425727	49425727	T	G	nonsynonymous SNV	NM_003482:exon39:p.Q4254P
125	chr12	49434697	49434697	C	A	stopgain	NM_003482:exon31:p.E2286X
125	chr12	49436086	49436086	T	G	nonsynonymous SNV	NM_003482:exon28:p.E1965D
125	chr12	49444885	49444885	A	C	nonsynonymous SNV	NM_003482:exon10:p.S861A
125	chr12	49444916	49444916	A	C	nonsynonymous SNV	NM_003482:exon10:p.H850Q
126	chr12	49420606	49420606	C	T	nonsynonymous SNV	KMT2D:NM_003482:exon48:c.G15143A:p.R5048H
126	chr12	49425724	49425724	C	A	nonsynonymous SNV	KMT2D:NM_003482:exon39:c.G12764T:p.G4255V
126	chr12	49426659	49426659	C	A	nonsynonymous SNV	NM_003482:exon39:p.Q3943H
126	chr12	49426660	49426660	T	A	nonsynonymous SNV	NM_003482:exon39:p.Q3943L
126	chr12	49445088	49445088	T	G	nonsynonymous SNV	NM_003482:exon10:p.E793A
127	chr12	49422963	49422963	G	-	frameshift deletion	KMT2D:NM_003482:exon44:c.14132delC:p.P4711fs
127	chr12	49418442	49418442	T	C	nonsynonymous SNV	KMT2D:NM_003482:exon50:c.A15971G:p.H5324R
128	chr12	49426916	49426916	G	A	stopgain	KMT2D:NM_003482:exon39:c.C11572T:p.Q3858X
128	chr12	49444726	49444726	C	A	stopgain	KMT2D:NM_003482:exon10:c.G2740T:p.E914X
129	chr12	49434114	49434114	-	A	frameshift insertion	KMT2D:NM_003482:exon31:c.7438_7439insT:p.K2480fs
129	chr12	49434115	49434115	T	A	stopgain	KMT2D:NM_003482:exon31:c.A7438T:p.K2480X
130	chr12	49439863	49439863	C	T	nonsynonymous SNV	KMT2D:NM_003482:exon17:c.G4678A:p.V1560M
130	chr12	49439883	49439883	C	A	nonsynonymous SNV	KMT2D:NM_003482:exon17:c.G4658T:p.C1553F
131	chr12	49426675	49426683	TGCTGCTGT	-	nonframeshift deletion	NM_003482:exon39:p.3935_3938del
131	chr12	49444849	49444849	G	A	stopgain	NM_003482:exon10:p.Q873X
132	chr12	49444270	49444270	G	C	nonsynonymous SNV	KMT2D:NM_003482:exon11:c.C3101G:p.P1034R
132	chr12	49446800	49446800	G	T	stopgain	KMT2D:NM_003482:exon8:c.C1010A:p.S337X
133	chr12	49434346	49434346	G	-	frameshift deletion	KMT2D:NM_003482:exon31:c.7207delC:p.L2403fs
133	chr12	49431094	49431094	T	C	nonsynonymous SNV	KMT2D:NM_003482:exon34:c.A10045G:p.M3349V
134	chr12	49420869	49420878	TCGGGCTGAT	-	frameshift deletion	KMT2D:NM_003482:exon48:c.14871_14880del:p.E4957fs
134	chr12	49431205	49431205	G	A	stopgain	KMT2D:NM_003482:exon34:c.C9934T:p.Q3312X

135	chr12	49416130	49416130	C	T	nonsynonymous SNV	KMT2D:NM_003482:exon52:c.G16345A:p.G5449S
135	chr12	49424495	49424496	AA	-	frameshift deletion	KMT2D:NM_003482:exon41:c.13727_13728del:p.F4576fs
136	chr12	49427714	49427714	T	G	nonsynonymous SNV	KMT2D:NM_003482:exon39:c.A10774C:p.M3592L
136	chr12	49434409	49434409	G	A	nonsynonymous SNV	KMT2D:NM_003482:exon31:c.C7144T:p.P2382S
137	chr12	49434409	49434409	G	A	nonsynonymous SNV	KMT2D:NM_003482:exon31:c.C7144T:p.P2382S
137	chr12	49431257	49431267	GGCCTGGGCAG	-	frameshift deletion	KMT2D:NM_003482:exon34:c.9872_9882del:p.P3291fs
138	chr12	49433006	49433006	C	T	nonsynonymous SNV	NM_003482:exon33:p.R2789Q
138	chr12	49434409	49434409	G	A	nonsynonymous SNV	NM_003482:exon31:p.P2382S
138	chr12	49447055	49447055	-	ATA	stopgain	NM_003482:exon7:p.G297delinsVX
139	chr12	49042089	49042089	C	T	nonsynonymous SNV	KMT2D:NM_003482:exon28:c.G6109A:p.D2037N
139	chr12	49032688	49032688	A	C	nonsynonymous SNV	KMT2D:NM_003482:exon39:c.T12017G:p.L4006R rs777667777
140	chr12	49031418	49031418	-	T	frameshift insertion	KMT2D:NM_003482:exon39:c.13286dupA:p.Q4429fs
140	chr12	49037283	49037283	C	-	frameshift deletion	KMT2D:NM_003482:exon34:c.10073delG:p.S3358fs
141	chr12	49024886	49024890	CGCAG	-	frameshift deletion	KMT2D:NM_003482:exon49:c.15841_15845del:p.L5281fs
141	chr12	49022588	49022588	A	C	splicing	KMT2D:NM_003482:exon51:c.16338+2T>G
142	chr12	49038898	49038898	G	A	stopgain	KMT2D:NM_003482:exon34:c.C8458T:p.Q2820X
142	chr12	49037331	49037331	C	T	nonsynonymous SNV	KMT2D:NM_003482:exon34:c.G10025A:p.R3342H
143	chr12	49031861	49031861	G	A	stopgain	KMT2D:NM_003482:exon39:c.C12844T:p.R4282X
143	chr12	49033482	49033485	TTGC	CTGT	nonframeshift substitution	KMT2D:NM_003482:exon39:c.11220_11223ACAG
